# Subarachnoid Hemorrhage From Rupture of an Undiagnosed Posterior Circulation Aneurysm During Sellar Tumor Surgery

**DOI:** 10.7759/cureus.21609

**Published:** 2022-01-25

**Authors:** Edgar Nathal, Rubén Mormandi, Andrés E Cervio, Juan P Navarro-Garcia de Llano, Alejandro Ceja-Espinosa

**Affiliations:** 1 Neurosurgery, Instituto Nacional de Neurología y Neurocirugía Manuel Velasco Suárez, Mexico City, MEX; 2 Neurosurgery, Instituto Fleni, Buenos Aires, ARG

**Keywords:** transsphenoidal surgery, posterior circulation aneurysm, ruptured cerebral aneurysm, subarachnoid hemorrhage, sellar tumor

## Abstract

Association between cerebral aneurysms and sellar tumors has been previously reported. Rupture of anterior circulation aneurysms during a transsphenoidal surgery causing massive subarachnoid hemorrhage (SAH) is uncommon, but rupture of a posterior circulation aneurysm is an infrequent event.

We present three cases of SAH secondary to rupture of an undetected posterior circulation aneurysm during transsphenoidal surgery to treat a sellar tumor. The common factor in these cases was the adverse outcome despite treatment.

The fatal outcome seen in all these cases questions whether to include a (magnetic resonance) MR angiography or (computed tomography) CT angiography during preoperative evaluation for sellar tumors in order to identify inadvertently associated aneurysms.

## Introduction

Pituitary tumors coexisting with cerebral aneurysms are a well-recognized association, but their frequency has not been definitively established.

Some studies report an incidence of 3.7%-7.4%, with 97% of the aneurysms located in the anterior circulation [1-4]. The occurrence of subarachnoid hemorrhage (SAH) caused by the rupture of a coexisting cerebral aneurysm is quite unusual, and SAH caused by the inadvertent rupture of a posterior circulation aneurysm during transsphenoidal surgery is out of the ordinary [5-9].

This work presents a case series of three patients operated on for a sellar tumor through a transsphenoidal route as scheduled cases. In all three, an unexpected sudden neurologic impairment established the diagnosis of a massive SAH, and rupture of a non-detected posterior circulation aneurysm on preoperative imaging studies was shown to be the cause.

## Case presentation

Case 1

**Figure 1:**
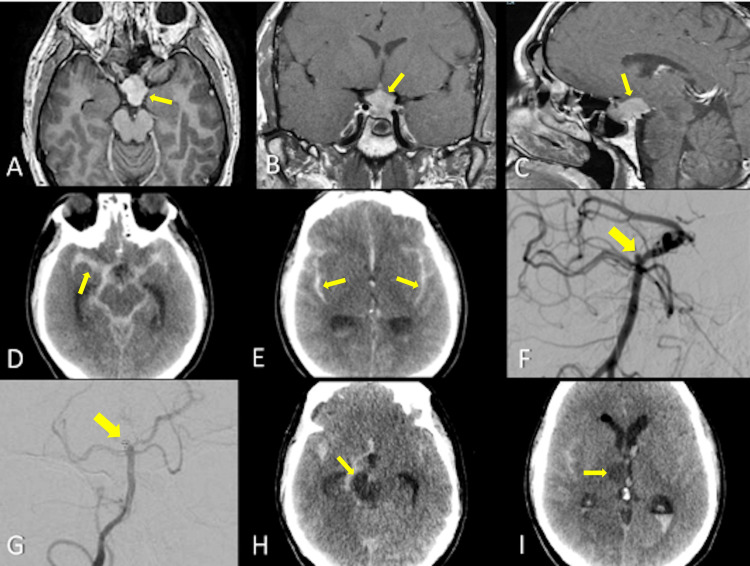
Case 1 representative MRI, CT, and DSA images. (A-C) MRI shows a sellar tumor compatible with pituitary adenoma bulging into the mesencephalic cistern (arrows). (D,E) PO CT displaying a massive subarachnoid hemorrhage extending into the peri-mesencephalic cisterns (arrows). (F) A small basilar top aneurysm was discovered during the DSA (arrow). (G) The aneurysm was coiled for dome protection (arrow). (H,I) CT at PO day 12. Low-density areas appeared at mesencephalic and thalamic areas (arrows). MRI, magnetic resonance imaging; CT, computed tomography; DSA, digital subtraction angiography; PO, postoperative.

This 38-year-old male presented occasional galactorrhea for the past six years. He started experiencing progressive visual loss two years ago, leading to the ophthalmologist who referred the patient to neurosurgical evaluation because bitemporal hemianopsia was found. Prolactin levels (PRL) were 3430 ng/mL, and the magnetic resonance imaging (MRI) showed a sellar/suprasellar mass bulging into the prepontine cistern and involving both cavernous sinuses (Figure 1A-1C). Medical treatment with cabergoline lowered PRL to 68 ng/mL, but with no shrinkage of the tumor and poor visual results, despite one year of pharmacologic treatment, making surgical removal necessary.

During the transsphenoidal approach, a sudden diaphragma sellae descent with systolic hypertension at 240 mm Hg and bradycardia prevented from continuing the procedure. A considerable pinkish secretion came out of the endotracheal tube, with a suspicion of pulmonary edema. Marked bilateral dilated pupils required an emergent unenhanced CT, revealing a severe SAH centered on the peri-mesencephalic cistern with reflux into the ventricles and moderate ventricular enlargement (Figure 1D-1E). After inserting an intracranial pressure monitor and an external ventricular drain placement for cerebrospinal fluid diversion, a catheter-based angiography disclosed a small top basilar aneurysm, later managed with coiling (Figure 1F-1G).

The postoperative course was complicated by diabetes insipidus, right middle cerebral artery vasospasm seen on transcranial insonation, and delayed cerebral ischemia. Diffuse infarcts from the right midbrain, thalamus, and cerebral hemisphere finally lead to the patient's death on day 15 (Figure 1H-1I).

Case 2

**Figure 2:**
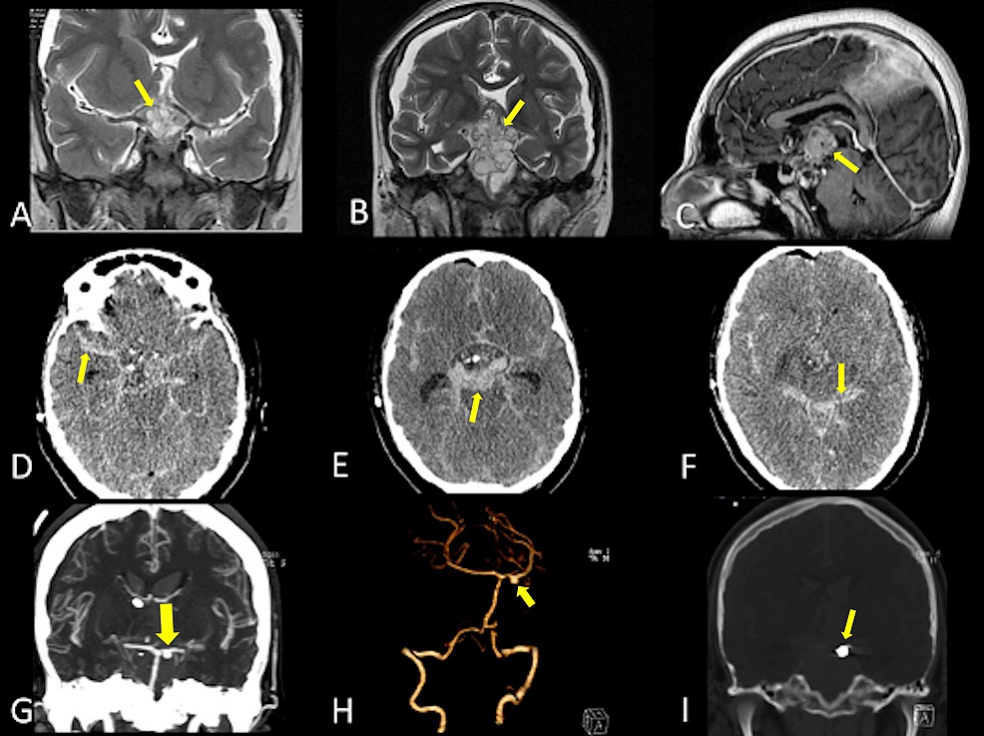
Case 2 representative MRI and CT angio images. (A-C) MRI shows a suprasellar lesion extending upwards. A retrosellar extension of the tumor is visible (arrows). (D-F) During surgery, severe bleeding was noticed and controlled. CT scan shows a generalized SAH with thick blood clots at the prepontine cistern (arrows). (G,H) CT angio shows a right small P1 aneurysm (arrows). (I) The aneurysm was treated by coiling (arrow). CT, computed tomography; MRI, magnetic resonance imaging; SAH, subarachnoid hemorrhage.

Six weeks before admission, this otherwise healthy 15-year-old male teenager initially presented with ocular pain, visual acuity impairment, frontal pulsatile exertional headache, recent nausea, and vomiting. The eye examination found papilledema, and loss of visual acuity on both eyes, while abduction of the left eye was limited. The imaging workup revealed a 43 × 27 × 28 mm sellar and suprasellar tumor, most likely a craniopharyngioma with secondary hypogonadotropic hypogonadism (Figure 2A-2C).

The patient was uneventfully managed with a right frontal ventriculoperitoneal (VP) shunt for obstructive hydrocephalus and a transsphenoidal resection. Heavy bleeding from the sellar area was controlled during surgery, but a postoperative CT revealed a Fisher 4 SAH (Figure 2D-2F). An external ventricular drain replaced the VP shunt. A CT angiography showed a left P1 aneurysm (5.4 × 5.3 mm with a neck of 3.8 mm) that was subsequently coiled with obliteration of 85% (Figure 2G-2I). Cerebral vasospasm in both anterior cerebral arteries and the left posterior cerebral artery marked a poor evolution, leading to anisocoria, bilateral fixed pupils, and loss of brain stem reflexes. The transcranial Doppler with a reverberant pattern confirmed the diagnosis of brain death, and the patient died two days later.

Case 3

**Figure 3:**
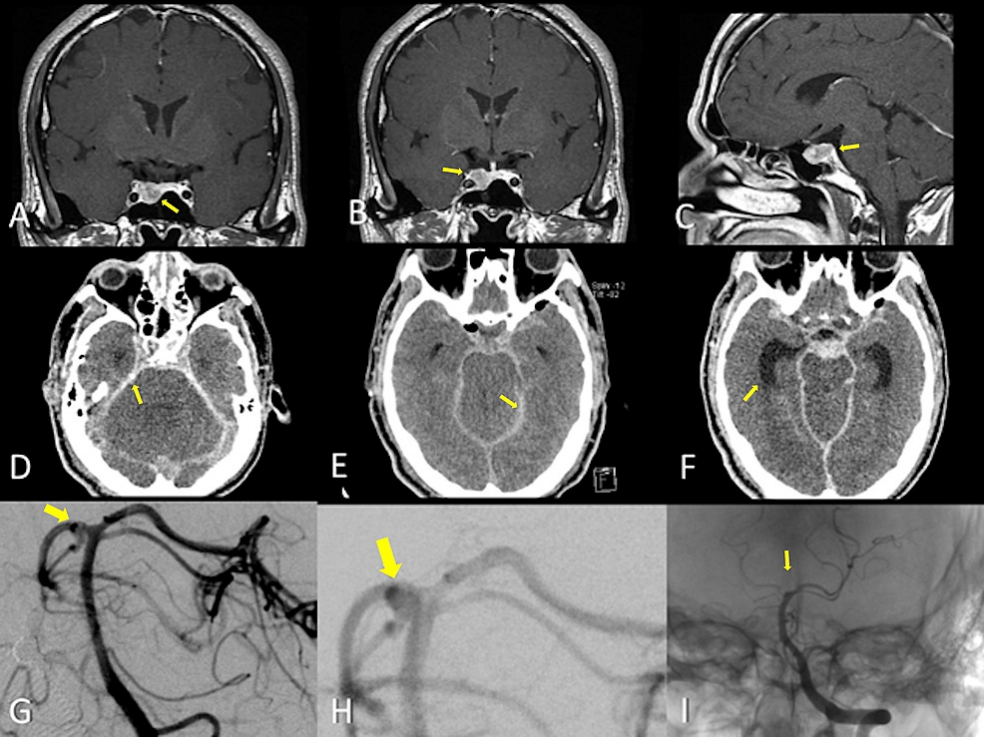
Case 3 representative MRI, CT, and DSA images. (A-C) MRI shows a sellar tumor without retrosellar extension (arrows). (D,E) PO CT scan showing a massive SAH filling all basal cisterns (arrows). (F) Early ventricular enlargement is seen (arrow). (G,H) A small basilar-superior cerebellar artery segment aneurysm is seen (arrow). (I) The patient was coiled for dome protection (arrow). CT, computed tomography; MRI, magnetic resonance imaging; DSA, digital subtraction angiography; PO, postoperative; SAH, subarachnoid hemorrhage.

A progressive visual impairment led this 45-year-old male to the ophthalmologist, who referred the patient with a typical chiasmatic visual field defect. The MRI study revealed a sellar lesion 1.8 × 1.0 × 1.5 cm in dimensions (Figure 3A-3C). As a nonfunctioning pituitary adenoma, he was taken to a transsphenoidal removal of the lesion with an unremarkable procedure. During emergence from anesthesia, the patient suddenly deteriorated with bilateral dilated and fixed pupils. A Fisher grade 3 SAH with obstructive hydrocephalus was shown by an unenhanced CT (Figure 3D-3F). After ventriculostomy placement, a 3-mm basilar aneurysm (Figure 3G-3H) at the superior cerebellar segment junction was diagnosed at digital subtraction angiography (DSA) and subsequently coiled for dome protection (Figure 3I). He progressively deteriorated until brain death and a final, fatal outcome.

## Discussion

Transsphenoidal surgery is one of the procedures of choice to remove pituitary adenomas and other sellar tumors, with mortality and morbidity rates below 0.5% and 1.5%, respectively [10]. Commonly reported complications are cerebrospinal fluid fistulas, pneumocephalus, neuroinfection, intracavernous carotid artery pseudoaneurysm formation, and traumatic SAH.

Cerebral aneurysms coexist with pituitary tumors more often than expected, and their presence on the major arteries adjacent to the pituitary and suprasellar tumors entails considerable risks, particularly when the aneurysm is closer to the operative field [1,11-13]. They are commonly detected at the imaging workup stage in preoperative MRI. It is, therefore, possible to plan the treatment of both lesions, separate or during the same procedure, particularly when the aneurysm is the area and is accessible from the same surgical approach [3]. Besides serendipity, another reason for explaining this association is the direct mechanical effect of sellar tumors on the vasculature, direct infiltration by tumor, and growth hormone production leading to arteriosclerosis, hypertension, and diabetes [1,2,8].

Previous reports locate most of these aneurysms in the anterior circulation, particularly in the anterior communicating or the internal carotid arteries, possibly because these arteries are related to the blood supply to the sellar region [4-6,11,13]. In one of these series, the aneurysm's location was posterior communicating segment of the internal carotid artery in 48%, ophthalmic segment in 19%, and carotid bifurcation in 13% [12]. In another study, 60% of the aneurysms were near the parasellar region and 40% away from it [2]. In these patients, the risk of aneurysmal rupture is always present. Some moments are particularly at risk: emergence from anesthesia or intraoperative manipulation to achieve a better tumor removal, direct mechanical impact, or intraoperative hemodynamic changes.

On the contrary, the occurrence with posterior circulation aneurysms is unusual and has been reported only twice. One was an autopsy case where a pituitary adenoma was associated with a giant vertebrobasilar aneurysm, and the other described a fatal SAH from a basilar apex aneurysm in the postoperative period [7,14]. Rupture of a posterior circulation aneurysm during craniopharyngioma removal has never been reported. These three cases are thus the largest series reporting the association of an undetected aneurysm of the posterior circulation associated with a sellar tumor. In all three cases, postoperative imaging documented a small posterior circulation aneurysm (top basilar, basilar artery-superior cerebellar artery segment, and P1 segment). Preoperative workup never disclosed clearly the presence of the aneurysm, and in contrast with reported cases regarding intraoperative rupture of anterior circulation aneurysms, all three patients had a fatal outcome [5,6,15,16]. Hemodynamic changes are less likely to occur in the posterior circulation while removing a pituitary adenoma or a craniopharyngioma compared with the anterior circulation. However, a retrosellar extension might be a risk factor for mechanical injury during tumor excision, even when the resection is made without incurring any extraordinary surgical maneuver.

Despite its rarity, postoperative SAH can have dismal consequences, as shown in this series, and it might deserve further consideration. Given its technical feasibility during the preoperative imaging workup without significantly increasing healthcare costs or scanning time, we wonder if complementary preoperative MR angiography or CT angiography should be performed, mainly when there is a mechanical involvement or closeness to vascular structures. Additionally, DSA should be mandatory when an associated aneurysm is detected preoperatively. In such cases, a better plan can be done including the dome protection through an endovascular route before the transsphenoidal surgery to avoid an eventual rupture. Another option is the treatment of both, the aneurysm and the adenoma, through a transcranial route at the same time.

Even when anterior circulation aneurysms are more commonly associated with pituitary adenomas (especially growth hormone-secreting tumors), posterior circulation aneurysms are not common in such situations. Focused preoperative imaging might be a way to prevent unforeseen catastrophic events. Moreover, this point remains controversial and demands further investigations.

## Conclusions

The coexistence of sellar and suprasellar tumors and posterior circulation aneurysms are rare. The occurrence of SAH after tumor removal is exceedingly exceptional and has rarely been reported. However, the fatal outcome of this unexpected event in an otherwise nonmortal disease deserves further study to determine if these cases could benefit from a more extensive workup vascular imaging in the preoperative stage.
